# High expression of the vacuole membrane protein 1 (*VMP1*) is a potential marker of poor prognosis in HER2 positive breast cancer

**DOI:** 10.1371/journal.pone.0221413

**Published:** 2019-08-23

**Authors:** Arsalan Amirfallah, Adalgeir Arason, Hjorleifur Einarsson, Eydis Thorunn Gudmundsdottir, Edda Sigridur Freysteinsdottir, Kristrun Audur Olafsdottir, Oskar Thor Johannsson, Bjarni Agnar Agnarsson, Rosa Bjork Barkardottir, Inga Reynisdottir

**Affiliations:** 1 Cell Biology Unit at the Pathology Department, Landspitali–The National University Hospital of Iceland, Reykjavik, Iceland; 2 The Biomedical Center, University of Iceland, Reykjavik, Iceland; 3 Molecular Pathology Unit at the Pathology Department, Landspitali–The National University Hospital of Iceland, Reykjavik, Iceland; 4 Pathology Department, Landspitali–The National University Hospital of Iceland, Reykjavik, Iceland; 5 Department of Oncology, Landspitali–The National University Hospital of Iceland, Reykjavik, Iceland; 6 Faculty of Medicine, University of Iceland, Reykjavik, Iceland; Taipei Medical University, TAIWAN

## Abstract

**Background:**

Fusion genes result from genomic structural changes, which can lead to alterations in gene expression that supports tumor development. The aim of the study was to use fusion genes as a tool to identify new breast cancer (BC) genes with a role in BC progression.

**Methods:**

Fusion genes from breast tumors and BC cell lines were collected from publications. RNA-Seq data from tumors and cell lines were retrieved from databanks and analyzed for fusions with SOAPfuse or the analysis was purchased. Fusion genes identified in both tumors (n = 1724) and cell lines (n = 45) were confirmed by qRT-PCR and sequencing. Their individual genes were ranked by selection criteria that included correlation of their mRNA level with copy number. The expression of the top ranked gene was measured by qRT-PCR in normal tissue and in breast tumors from an exploratory cohort (n = 141) and a validation cohort (n = 277). Expression levels were correlated with clinical and pathological factors as well as the patients’ survival. The results were followed up in BC cohorts from TCGA (n = 818) and METABRIC (n = 2509).

**Results:**

Vacuole membrane protein 1 (*VMP1*) was the most promising candidate based on specific selection criteria. Its expression was higher in breast tumor tissue than normal tissue (p = 1x10^-4^), and its expression was significantly higher in HER2 positive than HER2 negative breast tumors in all four cohorts analyzed. High expression of VMP1 associated with breast cancer specific survival (BCSS) in cohort 1 (hazard ratio (HR) = 2.31, CI 1.27–4.18) and METABRIC (HR = 1.26, CI 1.02–1.57), and also after adjusting for HER2 expression in cohort 1 (HR = 2.03, CI 1.10–3.72). BCSS was not significant in cohort 2 or TCGA cohort, which may be due to differences in treatment regimens.

**Conclusions:**

The results suggest that high VMP1 expression is a potential marker of poor prognosis in HER2 positive BC. Further studies are needed to elucidate how VMP1 could affect pathways supportive of tumorigenesis.

## Introduction

BC is the most common type of cancer diagnosed in women worldwide [1]. The prognosis and treatment depend on the stage of the disease at diagnosis, the type of tumor, the grade, the proliferation status (Ki67 expression), and the expression of HER2/ERBB2 and the hormonal receptors, estrogen and progesterone receptors. Even though drugs, which are tailored to the genetic make-up of a tumor such as HER2 expression, are increasingly being used, not all tumors respond to treatment and options for further targeted treatment is limited for patients that experience relapse of their disease. Therefore, identifying new genes that support tumor progression in the breast could be used to improve prognosis and follow-up of patients.

Genes that support tumorigenesis most often have undergone changes that result in loss of control or changes in expression patterns. Genetic rearrangements such as amplification, translocations, inversions, insertions and deletions are frequent in breast tumors. Amplified chromosomal regions are well known in breast tumors [2], particularly the amplifications of the *ERBB2* locus at 17q12. It results in the gene´s overexpression giving the cell the potential to bypass regulatory mechanisms and support malignant growth. Fusion genes, generated through inter-chromosomal translocations or intrachromosomal changes such as inversions or deletion of chromosomal segments, also can acquire such oncogenic potential [3, 4]. Recurring fusion genes have only been identified in subgroups of breast tumors [5–7] rather than across different subtypes of breast tumors [4, 8]. Most studies have focused on functional chimeric fusion proteins even though they are a minority of fusion genes [4, 8, 9]. Translocations can result in inappropriate expression of genes through promoter switching [10] and loss of 3´ UTR regulation by miRNAs [5, 11]. They can activate intragenic miRNAs inappropriately [5] as well as place superenhancers in the vicinity of genes resulting in overexpression of genes in the absence of amplification [12]. As such, a genetic rearrangement that results in a fusion gene may produce a single gene with malignant properties rather than produce a functional chimera made from two genes. Therefore, we postulated that screening fusion genes could be used as a tool to identify potentially novel cancer genes that can affect tumor development. Herein, we describe a screen of fusion genes in a large group of breast tumors and in BC cell lines that identified vacuole membrane protein 1 (*VMP1*) as a gene that may contribute to breast tumor progression.

## Materials and methods

### Fusion genes from breast tumors and BC cell lines

Fusion genes from breast tumors were collected from three studies [4, 9, 13]. In addition, a list of fusion genes from breast tumors from The Cancer Genome Atlas (TCGA) was purchased from MediSapiens (www.medisapiens.com). They used the MediSapiens FusionSCOUT pipeline to identify fusion genes in RNA-Seq data. Fusion genes from BC cell lines were collected from publications [14–20]. Furthermore, we analyzed RNA-Seq data from BC cell lines with the fusion finding algorithm SOAPfuse [21]: CAMA-1 (GSM1172856), MDAMB134VI (GSM1172886), MDA-MB-231 (GSM1172889), SUM-225 (GSM1172901), SUM-229 (GSM1172902), SUM52 (GSM1172903), SUM44 (GSM1897347), and UACC893 (GSM1172907/GSM1897353). The paired-end RNA-Seq data from the cell lines were mapped to the human reference genome (hg19) and annotated transcripts (Ensembl release 75) using SOAP2. Then, SOAPfuse was used to identify fusion genes by detecting span and junction reads from the aligned data. Analyses of the RNA-Seq data from the cell lines also were purchased from MediSapiens. Fusion genes in BC cell lines that were identified by both MediSapiens FusionSCOUT pipeline and SOAPfuse were considered for validation.

### Cohorts and tissue samples and clinical data

Cohort 1 consisted of 158 BC patients, diagnosed 1987–2003 [22], and cohort 2 consisted of 291 patient, diagnosed 2003–2007 (S1 Table). The relevant patient data were collected from hospital records at Landspitali–The National University Hospital of Iceland as described previously [22]. Primary fresh frozen tumors were obtained from the Department of Pathology as well as six non-neoplastic breast tissue, taken as far away from the tumor as possible. Informed consent was obtained from all patients involved in this study according to the national guidelines. The study was approved by The Icelandic Data Protection Commission (2001/523 and 2002/463) as well as the National Bioethics Committee of Iceland (99/051, 99/051_FS1, VSN-11-105, VSN-15-138). The Nordic cohort consisted of 577 primary breast tumors from patients whose majority was diagnosed 1987–2003 in Finland, Sweden and Iceland (including samples from cohort 1) [23, 24]. TCGA cohort consisted of 818 BC patients diagnosed 1988–2013 [25] and the METABRIC patients were 2,509, diagnosed 1980–2005 [26–28], with data available for both cohorts through cBioPortal [29, 30] and from Rueda et al. [28].

### DNA and RNA isolation

DNA and total RNA were extracted from fresh frozen breast tumors from patients in cohort 2 (n = 291) and from 6 normal breast tissue samples as well as 1x10^6^ MCF-7 cells using Allprep kit DNA/RNA/miRNA (Qiagen no. 80224) according to protocol. The extraction from cohort 1 has been described [22] but in short, total RNA was extracted with Trizol and purified on an RNeasy column according to protocol. The quantity of DNA was measured by Nanodrop 1000 and the RNA quality was measured with Bioanalyzer 2100 RNA 6000 Nano kit (Agilent Technologies, cat. no. 5067–1511) according to protocol. The majority of tumors had RIN ≥ 8.

### Verification of RPS6KB1-VMP1 in MCF-7

MCF-7 was obtained from the American Type Culture Collection. It was cultured in DMEM/F12 (ThermoFisher, cat.no. 11330–032) supplemented with 10% fetal calf serum (ThermoFisher, cat.no. 10270–106), 37°C and 5% CO_2._ RNA was extracted as described above and cDNA was synthesized using a RevertAid First Strand cDNA Synthesis Kit (Thermo Fisher Scientific no. 1622). The RPS6KB1-VMP1 junction was amplified by PCR and then sequenced using primers F: 5´-GAAACTAGTGTGAACAGAGG-3´ and R: 5´-CATAACTTTGTGCCATGGAG-3´.

### *VMP1* copy number variations

*VMP1* copy number data from the Nordic dataset were retrieved from GEO dataset GSE22133 [31] and from the TCGA dataset through cBioPortal [29, 30]. Both sets were measured by comparative genomic hybridization (CGH) on microarrays. The definition of copy number variation (CNV) in the TCGA dataset was used [32].

### VMP1 mRNA expression

VMP1 mRNA data for the Nordic dataset were retrieved from GEO (dataset GSE25307) and for the TCGA dataset through cBioPortal [29, 30]. Both sets were measured with gene expression microarrays with probes located at the 3’ end of VMP1. Total RNA (0.5 μg) from normal breast tissue and the tumors from cohorts 1 and 2 was used as a template to generate cDNA as described above. Quantification of the VMP1 mRNA level was performed with Taqman Gene Expression Assays spanning exons 10–11 (E10-11; Thermo Fisher Scientific, Taqman /Hs00978589_m1) in both cohorts, and a probe spanning exons 2 and 3 (E2-3; Taqman/Hs00978582_m1) was used to verify the data for cohort 1. TATA-binding protein (TBP, 1702071 Applied Biosystems) was used as a reference gene. All reactions were done in triplicate using 42 cycles with one ng of cDNA as template. VMP1 expression was calculated relative to TBP: 2^-(mean Ct target–mean Ct reference)^. mRNA values were obtained from 141 and 277 tumors in cohorts 1 and 2, respectively. The location of the VMP1 probes is shown in S1 Fig.

### Quantification of miR21 expression

cDNA synthesis for miRNA was performed using cDNA synthesis kit II (Exiqon cat. no. 203301) according to the manufacturers protocol. Five ng/μl of RNA from cohort 1 (n = 144) were used. The qRT-PCR reaction was performed with EXIQON primer sets hsa-miR21-5P (YP00204230) and hsa-miR21-3P (YP00204302) along with ExiLENT SYBR Green master mix and hsa-miR16-5P (YP00205702) as reference gene. All reactions were done in triplicate using 40 cycles.

### Statistical analysis

The statistical program R version 3.4.3 was used [33]. The microarray DNA and mRNA measurements from the Nordic dataset as well as the DNA, mRNA and miRNA measurements from cohorts 1 and 2 were transformed with log2 to normalize the data. The mRNA values from the METABRIC and TCGA cohorts, available from cBioPortal, are Z-scores. Co-amplification of *ERBB2* and *VMP1* DNA levels was analyzed with χ^2^-test. Correlation between DNA and mRNA levels, or mRNA and miRNA expression, was performed by calculating the Pearson correlation coefficient using normalized values. The correlation analyses between mRNA levels and the clinicopathological characteristics were performed with Student´s t-test or ANOVA. P-values below 0.05 were considered significant.

The Kaplan-Meier and log rank test were used to estimate survival using the survival package and the survminer package in R. Survival analysis was based on tumor VMP1 mRNA levels measured by microarrays in the Nordic (n = 553), TCGA (n = 421), and METABRIC (n = 1904) cohorts, and by qPCR with probe E10-11 in cohorts 1 (n = 141) and 2 (n = 277). The tumors were classified as expressing high VMP1 mRNA (≥ mean + 1 SD) or normal VMP1 mRNA (< mean + 1 SD). Hazard ratio (HR) calculation based on VMP1 mRNA levels and clinicopathological characteristics was performed with Cox regression analysis [34]. Due to missing data for VMP1 mRNA as well as lack of complete clinical data in some cohorts the numbers of patient samples in the analyses are lower than the actual number of patients.

## Results

### A screen of fusion genes identifies *VMP1* as a candidate

The generation of fusion genes may lead to loss of control and affect expression of the gene partners. We wanted to explore whether the genes that constitute fusion genes could be used to detect a gene that supports breast cancer development. Therefore, a screen of fusion genes was performed. It entailed the comparison of fusion genes, identified in breast tumors, with fusion genes identified in breast tumor cell lines. Cell lines tend to be aggressive and we reasoned that studying them would increase the likelihood of detecting a gene which is significant in the progression of BC. Fusion genes from BC cell lines were collected from publications [14–18, 20, 35] and RNA-Seq data were analyzed by fusion finding algorithms [21] as described in methods. Information regarding fusion genes and potential fusion genes from breast tumors were acquired from three studies [4, 9, 13] or from MediSapiens. In all, 183 fusion genes (paired genes) were acquired from 45 BC cell lines while 5319 fusion genes were acquired from 1724 breast tumors. The tumors and the cell lines had 15 fusion genes in common. They had to meet the following criteria to merit further analyses: 1) have a similar breakpoint in breast tumors and cell lines, 2) be recurrent in tumors, 3) not be located within an amplicon carrying a known oncogene unless it was part of the fusion, and 4) possess a function supportive of tumorigenesis (available through publications). Five fusion genes met these criteria (Table 1). They were all verified by PCR-amplification and sequencing in their respective cell lines (S2 Fig).

**Table 1:** Five fusion genes in common between breast tumors and breast cancer cell lines.

5´fusion gene partner	3´fusion gene partner	No of fusions (%)	Cell lines
CCDC6	ANK3	2 (0.12)	UACC893
ESR1	CCDC170	11 (0.64)	ZR751
GATAD2B	NUP210L	1 (0.06)	MCF-7
ITGB6	RBMS1	1 (0.06)	UACC893
RPS6KB1	VMP1	5 (0.29)	MCF-7

Fusion genes were analyzed in a total of 1724 breast tumors. The number of breast tumors carrying the fusion genes that were found in common between breast tumors and breast cell lines is shown in this table. The common fusions were analyzed in other tumor types through this website: www.tumorfusions.org [4]. GATAD2B-NU210L appeared once in these tumor types: uterine carcinosarcoma (UCS), lung adenocarcinoma (LUAD) and ovarian tumors (OV). ITGB6-RBMS1 was found in two tumors from bladder cancer (BLCA).

To distinguish which of the 10 genes that constituted the five fusions could be of consequence in BC progression, the copy number of the genes was analyzed and correlated with the respective mRNA levels. CGH microarray and gene expression data from our earlier study on 577 Nordic tumors [24] were used as well as data retrieved from TCGA [25]. Genes that are amplified and with highly correlating gene expression can signify an oncogene. Correlation between DNA and mRNA was highest for *CCDC6* (r = 0.66), *GATAD2B* (r = 0.54), *RPS6KB1* (r = 0.83) and *VMP1* (r = 0.70) in the cohort from TCGA (S2 Table). *CCDC6* was not amplified and even though *GATAD2B* was amplified in the TCGA cohort it was not amplified in the Nordic cohort. *RPS6KB1* and *VMP1* were the genes most frequently amplified in both cohorts, with amplification close to 11% in tumors from TCGA (S2 Table). *RPS6KB1* and *VMP1* are adjacent genes at 17q23. A tandem duplication of the locus was found in MCF-7 cells that resulted in a fusion between *RPS6KB1* and *VMP1* [36]. Although the tandem duplication was not common, they observed the *RPS6KB1-VMP1* fusion transcript, with varying breakpoints, in 22 tumors from a cohort of 70 BC patients from Singapore [36]. The fusion was observed in only five of 1724 tumors in our study (Table 1), only one of which was HER2 positive. The discrepancy in the frequency could be due to the ethnicity of the patients, from Singapore [36] as opposed to cohorts in which the majority of patients were of European descent [8, 26, 27], or it could be due to the method, specific screening for the *RPS6KB1-VMP1* fusion [36] as opposed to searching for fusion genes using RNA-Seq data, which was the basis of our study. The *RPS6KB1-VMP1* fusion was not enriched in HER2 positive tumors in the data that we used (one in five tumors) and of the 45 cell lines that we used it was only found in MCF-7, which is HER2 negative. Again, the depth of RNA sequencing and different fusion finding algorithms used in the various studies may be the reason. Interestingly, *VMP1* was found as a 3´ partner in fusion transcripts in 16 of the 1724 tumors (0.93%). Four of the tumors with VMP1 fusions, or 25%, were HER2 positive while 16% of the tumors with non-VMP1 fusions were HER2 positive. This is in accordance with Persson et al. [5], who showed VMP1 fusion transcripts to be enriched among HER2 positive tumors. Interestingly, the majority of the in frame fusion transcripts identified by Inaki et al. [36] included only the first exon of *RPS6KB1* and the C-terminal half of *VMP1*. Thus, the functional activity of the chimeric protein would be expected to stem from VMP1. In addition, *RPS6KB1* has been shown to associate with HER2 positivity and a worse outcome in BC ([37] and references therein). Thus, *VMP1* was selected as a candidate.

### *VMP1* is a potential player in breast tumorigenesis

**Fig 1:**
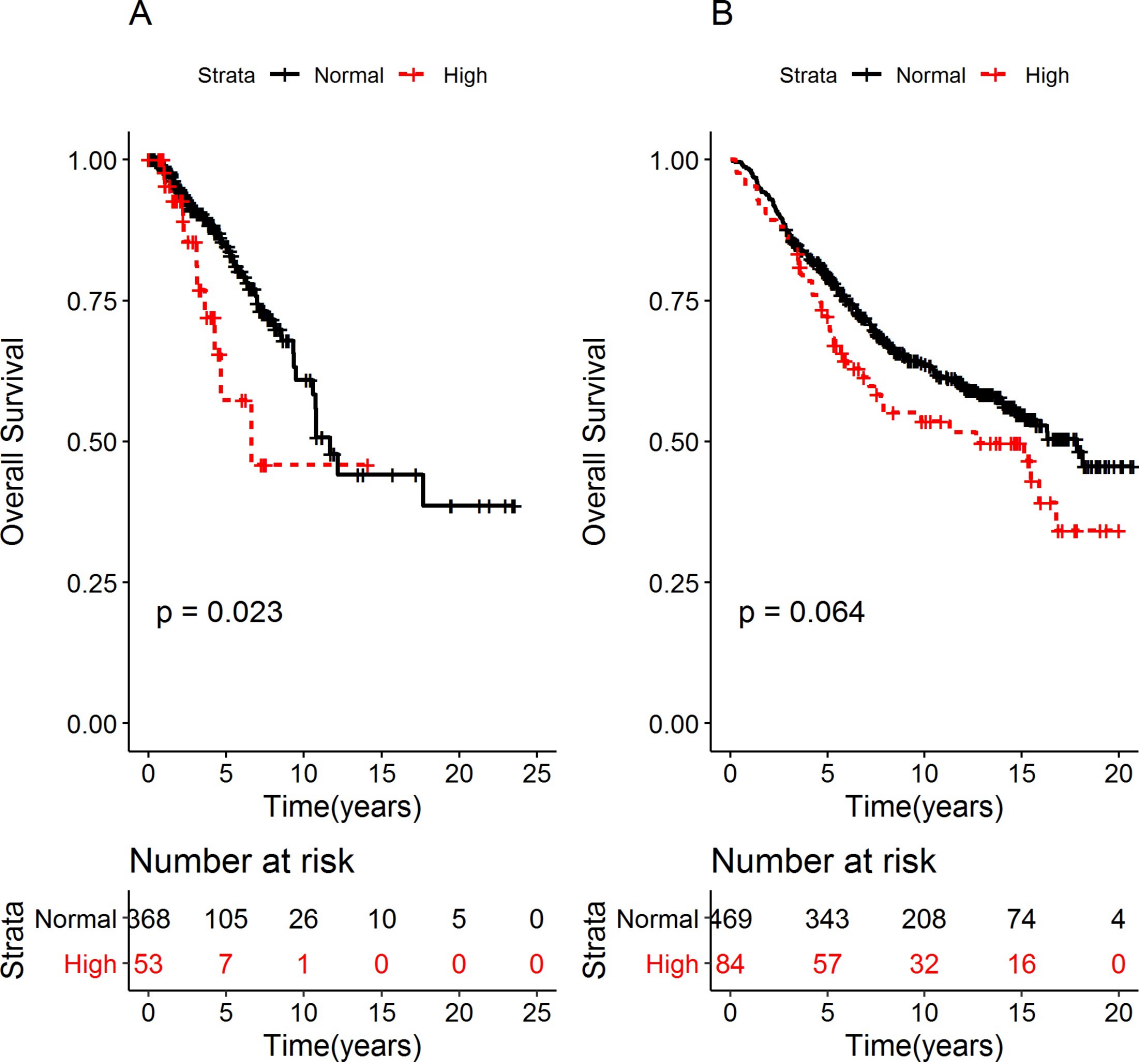
High VMP1 mRNA expression is correlated with shorter OS. Overall survival (OS) was examined in breast cancer patients in (A) TCGA and (B) the Nordic cohort. The patients were divided into two groups according to VMP1 mRNA levels: tumors expressing high VMP1 mRNA (high ≥ mean + 1 SD) and normal VMP1 (normal < mean + 1 SD). The log rank p-values are indicated in the graphs. The number of patients at risk is shown below the graphs in tables at the indicated time points. The median OS for the TCGA cohort was 11.68 and 6.62 years for patients expressing normal and high levels of VMP1 mRNA, respectively. The hazard ratio (HR) for OS was 2.10, CI 1.09–4.04The median OS in the Nordic cohort was 16.3 and 12.6 years for patients expressing normal and high VMP1 mRNA, respectively. The HR was 1.37, CI: 0.98–1.91.

Further analyses were performed in the Nordic and TCGA cohorts to explore the potential role of *VMP1* in breast tumor development. The highest correlation between *VMP1* DNA and mRNA was observed in tumors with *VMP1* amplification (TCGA: r = 0.72, p = 3.4x10^-9^) and in tumors with overexpression of *ERBB2*, either according to molecular subtype [38] (Nordic cohort: r = 0.79, r = 4.31x10^-8^) or HER2 expression (TCGA: r = 0.82, p = 2.2x10^-16^). To examine whether there were consequences of high VMP1 expression, survival analyses were performed in the Nordic and TCGA cohorts. They suggested shorter overall survival (OS) in BC patients carrying tumors with high levels of VMP1 mRNA (TCGA: log rank p-value = 0.023 and Nordic: log rank p = 0.064, Fig 1). The hazard ratio (HR) was 2.10 (CI 1.09–4.04) in TCGA and 1.37 (CI 0.98–1.91) in the Nordic cohort. One indication of oncogenic properties of a gene is higher expression levels in tumors than normal tissue. To compare expression in our cohorts, RNA was extracted from tumors in cohorts 1 (n = 141) and 2 (n = 277), and from the available normal breast tissue samples (n = 6) from cohort 2. VMP1 mRNA was measured by qPCR. It was found to be significantly higher in breast tumors from cohort 1 (p = 1x10^-4^) and cohort 2 (p = 3x10^-4^) than in normal breast tissue (S3 Fig).

VMP1 is located at 17q23, a chromosomal region whose copy number is increased in up to 22% of primary breast tumors depending on their histological origin [39]. Many genes reside within the amplified region but *RPS6KB1*, *MIR21* [40], and *PPMD1* [41] have been suggested as drivers of the amplification with oncogenic properties. In the TCGA cohort, VMP1 mRNA positively correlated with the mRNAs of RPS6KB1 (r = 0.67, p <2.2x10^-16^) and PPMD1 (r = 0.58, p < 2.2x10^-16^), and with miR21 (r = 0.50, p = 1.75x10^-12^). As expression from these genes could affect survival on their own and thus confound the effect observed with VMP1, a Cox regression analysis was performed to adjust for their expression. Expression of these genes did not attenuate the effect of VMP1 mRNA on OS in the TCGA cohort (S3 Table).

*MIR21* overlaps the 3´ end of *VMP1* [42, 43], and many of the fusion gene breakpoints within *VMP1* occur just prior to MIR21 (http://www.tumorfusions.org/, [5, 36]). The probes used to measure VMP1 mRNA in TCGA and cohorts 1 and 2 (spanning E10-11) were located in the C-terminus and potentially can detect pri-miRNA-21. Thus, VMP1 mRNA was measured with a probe spanning E2-3 in cohort 1, and the expression of the mature miRNA products, hsa-mir-21-5p and hsa-mir-21-3p was measured as well. The correlation between the VMP1 E2-3 and E10-11 probes was high (r = 0.85, p < 0.001). The VMP1 mRNA probes did not correlate with hsa-mir-21-5p or hsa-mir-21-3p (p > 0.05), indicating that the signal from the E10-11 probe reflected VMP1 mRNA levels.

Taking the data together, they suggest that *VMP1* may have oncogenic properties, and we wanted to explore whether VMP1 mRNA levels could have a prognostic value.

### VMP1 mRNA level is high in breast tumors that express HER2

**Fig 2:**
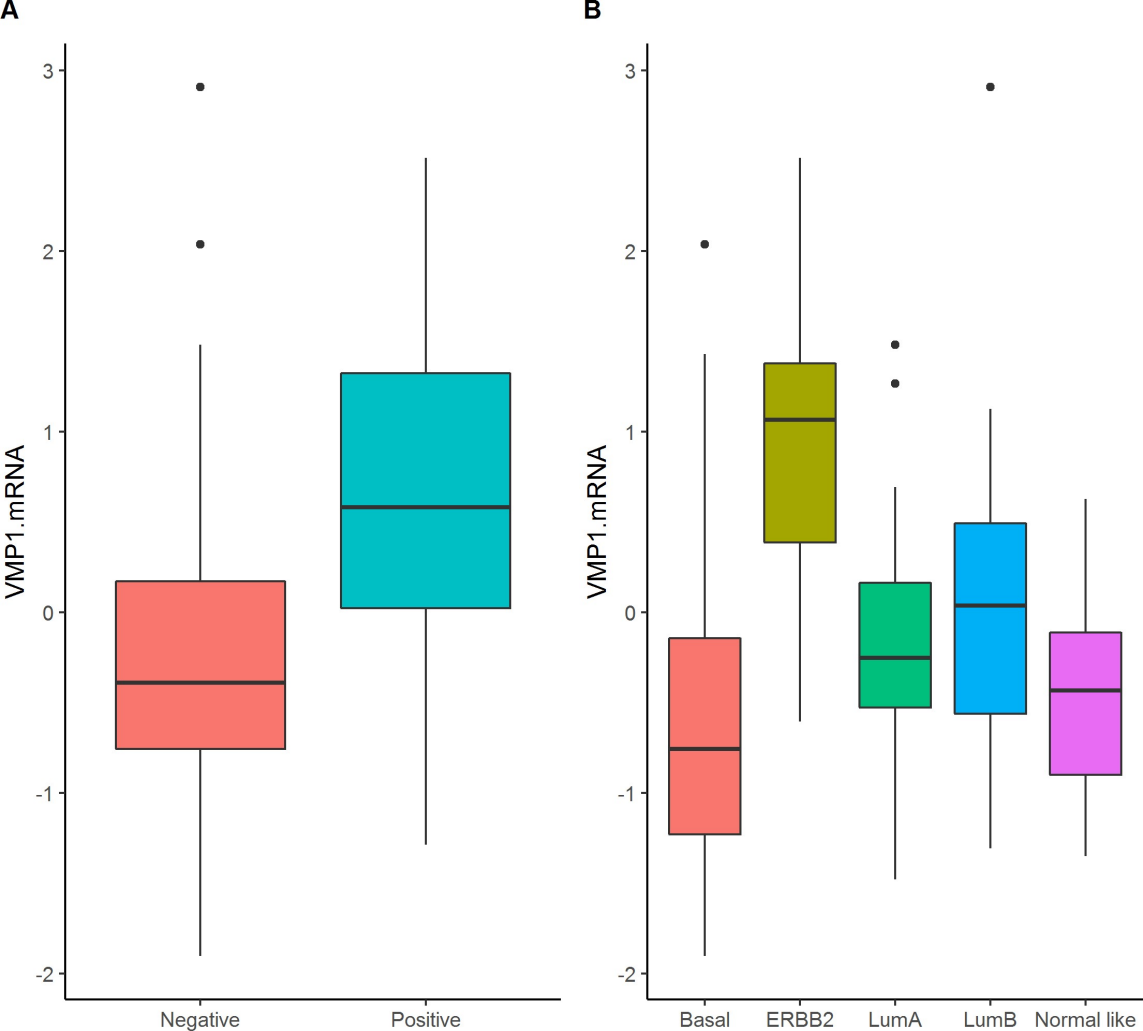
VMP1 mRNA is higher in HER2 positive and ERBB2 breast tumor subtype. VMP1 mRNA was examined according to (A) HER2 expression and (B) molecular subtype in breast tumors from cohort 1. VMP1 mRNA levels were compared between HER2 positive (n = 23) and HER2 negative (n = 117) tumors, and according to the molecular subtypes (basal = 24, ERBB2 = 14, luminal A = 43, luminal B = 30, normal-like = 12). The p-value was calculated with values normalized by log2 using a t-test for HER2 expression and ANOVA for the molecular subtypes.

In order to understand whether VMP1´s expression levels could indicate severity of disease the mRNA values were correlated with the tumor´s clinical and pathological characteristics. In breast tumors from cohort 1, higher VMP1 expression level was observed in ERBB2/HER2 positive tumors based on classification with immunohistochemistry (HER2, p = 7x10^-4^) or molecular subtyping (p = 5x10^-6^, Fig 2 and Table 2, [38]). There was a highly significant association between HER2 positivity and increased VMP1 mRNA levels in all cohorts: in cohort 2 p = 0.004 (S4 Table), in TCGA p = 0.003 (S5 Table), and in METABRIC p < 2.2x10^-16^ (S6 Table). The significant association between VMP1 mRNA levels and the intrinsic subtypes in TCGA and METABRIC were due to high levels of VMP1 mRNA in ERBB2 and luminal B subtypes, which include HER2 positive tumors, and low levels of VMP1 mRNA in the basal subtype, which reflected low VMP1 expression in ER negative tumors (TCGA: p = 7x10^-6^ and METABRIC: p = 0.01). This result was supported at the genomic level since the loci hosting *ERBB2* (17q12) and *VMP1* (17q23) were frequently co-amplified as has been published [23, 44] and seen in the Nordic cohort (χ^2^ test <2.2x10^-16^). The linear correlation between the CNVs of *ERBB2* and *VMP1* was low (CNV r = 0.28, p = 4.6x10^-7^) as well as between their mRNA (r = 0.30, p = 7.810^−14^) indicating that VMP1 expression was not high in all *ERBB2* amplified or highly expressing tumors. The data show that VMP1 is highly expressed or amplified in some HER2 positive or *ERBB2* amplified tumors, which may indicate a potential interaction between the two genes.

**Table 2:** Correlation of VMP1 mRNA with clinicopathological characteristics of breast tumors in cohort 1.

Characteristic	n = 141	VMP1 mRNA level median (25^th^, 75^th^)	p-value
Age			
≥ 50	85	-0.23 (-0.59, 0.28)	0.67
< 50	56	-0.28 (-0.78, 0.57)	
Estrogen receptor			
positive	90	-0.20 (-0.59, 0.32)	0.41
negative	47	-0.39 (-1.03, 0.50)	
unknown	4		
Progesterone receptor			
positive	70	-0.19 (-0.57, 0.43)	0.42
negative	64	-0.31 (-0.97, 0.37)	
unknown	7		
HER2 status			
positive	23	0.58 (0.02, 1.26)	7x10^-4^*
negative	117	-0.36 (-0.75, 0.18)	
unknown	1		
Receptors ER and HER2			
ER^-^ and HER2^-^	32	-0.56 (-1.14, -0.10)	6.4x10^-6^*
ER^-^ and HER2^+^	14	0.51 (-0.13, 1.20)	
ER^+^ and HER2^-^	82	-0.24 (-0.59, 0.23)	
ER^+^ and HER2^+^	8	0.48 (0.29, 1.25)	
unknown	5		
Tumor size (mm)			
> 20	97	-0.31 (-0.69, 0.40)	0.90
≤ 20	44	-0.14 (-0.62, 0.31)	
Histological type			
IDC^a^	121	-0.14 (-0.65, -0.43)	0.24
ILC^b^	12	-0.35 (-0.56, -0.13)	
other	8	-0.37 (-0.74, -0.10)	
Nodal status			
positive	72	-0.20 (-0.55, 0.57)	0.16
negative	55	-0.13 (-0.88, 0.38)	
unknown	14		
Ki 67			
High	41	-0.46 (-0.85, 0.28)	0.15
Low	97	-0.17 (-0.53, 0.51)	
Unknown	3		
Histological grade			
1	12	-0.49 (-0.67, 0.26)	0.08
2	80	-0.15 (-0.52, 0.25)	
3	48	-0.25 (-0.80, 0.78)	
unknown	1		
Metastasis			
Positive	59	-0.14 (-0.52, 0.60)	0.03*
Negative	81	-0.36 (-0.75, 0.25)	
unknown	1		
Intrinsic subtype			
Basal	24	-0.75 (-1.22, -0.14)	5x10^-6^*
ERBB2	14	0.82 (0.37, 1.35)	
Luminal A	43	-0.24 (-0.51, 0.17)	
Luminal B	30	0.03 (-0.56, 0.49)	
Normal-like	12	-0.47 (-0.87, -0.17)	
unknown	18		
Familial status			
BRCA2	27	-0.43 (-0.89, 0.39)	0.31
Non-BRCA2	114	-0.20 (-0.64, 0.38)	

The table shows the median and the 25^th^ and 75^th^ percentiles. One tumor was BRCA1 positive and it was not used in the familial status calculations. The p-value is calculated with log_2_ transformed data using a t-test or ANOVA.

*Significant difference p < 0.05.

^a^IDC: Invasive ductal tumors.

^b^ILC: Invasive lobular tumors.

### High VMP1 mRNA is associated with shorter survival

**Fig 3:**
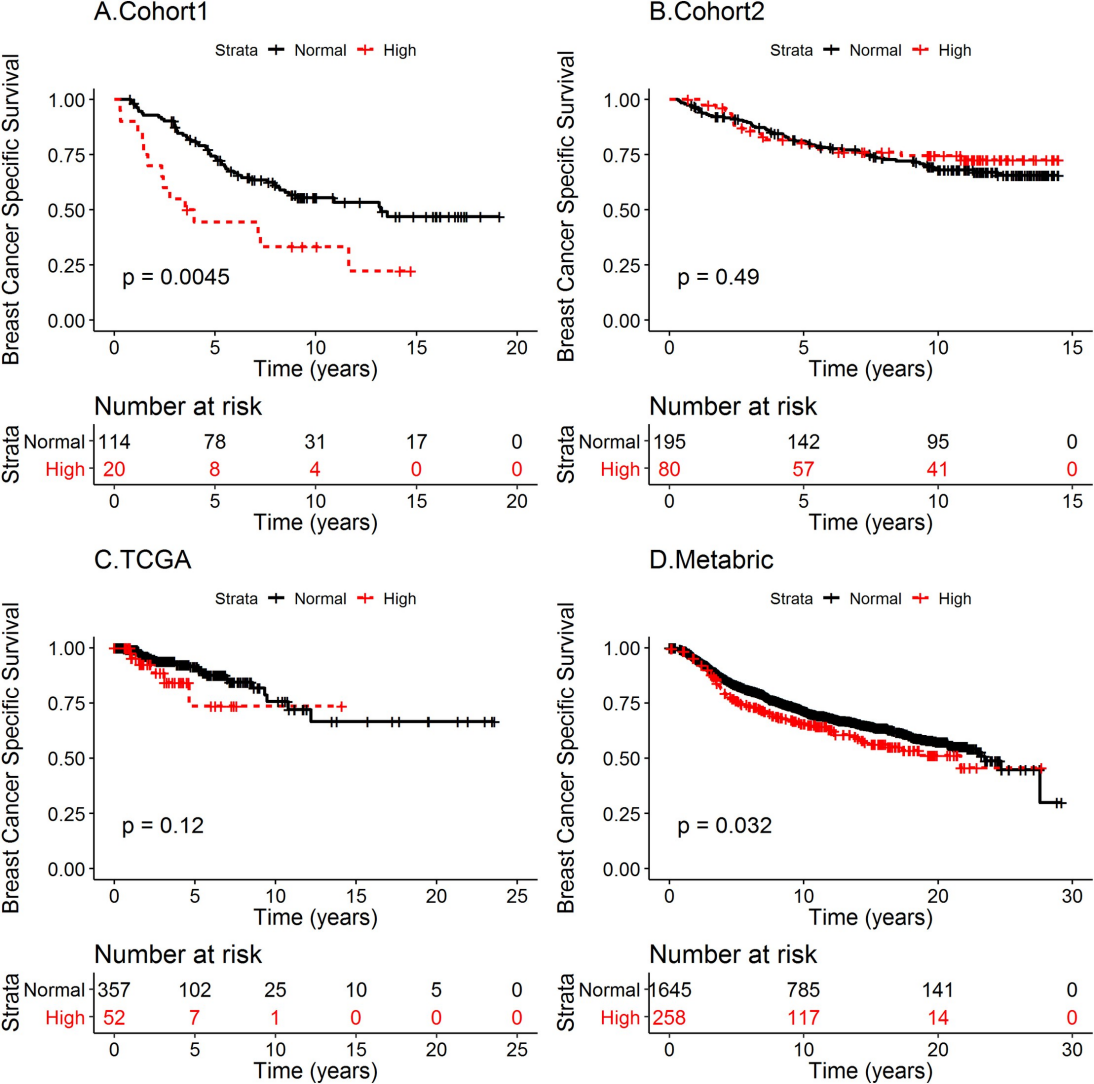
High VMP1 mRNA expression is correlated with shorter BCSS in cohort 1 and METABRIC. Breast cancer specific survival (BCSS) was analyzed in (A) cohort 1, (B) cohort 2, (C) TCGA and (D) METABRIC. The patients were divided into two groups according to VMP1 mRNA levels: tumors expressing high VMP1 mRNA (high ≥ mean + 1 SD) and normal VMP1 (normal < mean + 1 SD). The log rank p-values are indicated in the figures. The number of patients at risk is shown below the graphs at the indicated timepoints. The median BCSS was 13.22 and 3.75 years for patients expressing normal and high VMP1 mRNA, respectively, in cohort 1, and 23.5 and 21.7 years in the METABRIC cohort. The hazard ratio (HR) for BCSS in cohort 1 was 2.31 (CI 1.27–4.18), and after adjusting for HER2 expression the HR was 2.03 (CI 1.00–3.72). In METABRIC HR was 1.26 (CI 1.02–1.57) and after adjusting for HER2 expression it was HR = 1.03 (CI 0.82–1.30).

**Fig 4:**
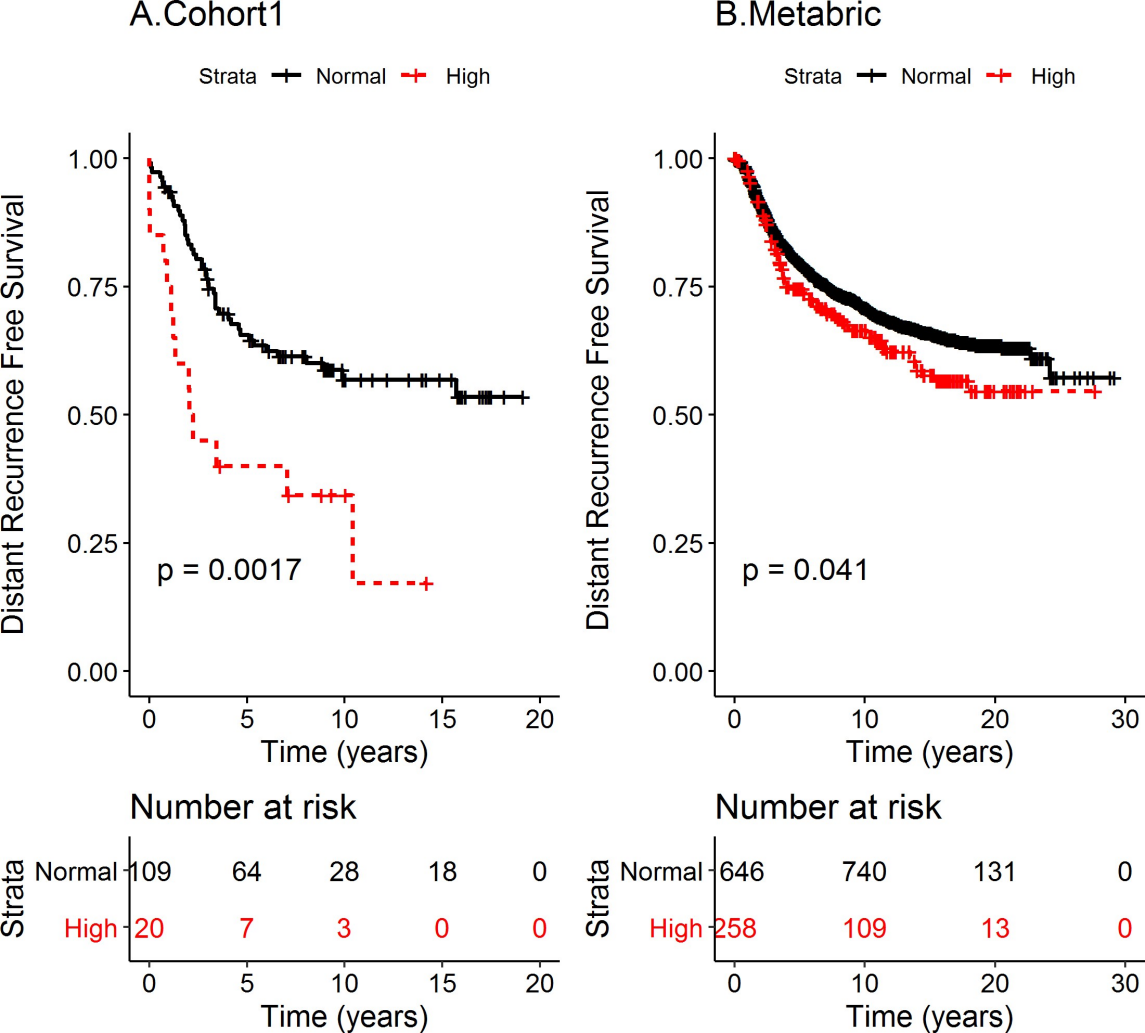
High VMP1 mRNA expression is correlated with shorter DRFS in cohort 1 and METABRIC. Distant recurrence free survival (DRFS) was analyzed in (A) cohort 1 and (B) METABRIC. The patients were divided into two groups according to VMP1 mRNA levels: tumors expressing high VMP1 mRNA (high ≥ mean + 1 SD) and normal VMP1 (normal < mean + 1 SD). The log rank p-values are indicated in the figures. The number of patients at risk is shown below the graphs at the indicated time points. The hazard ratio (HR) for DRFS in cohort 1 was 2.54, CI (1.39–4.66), and after adjusting for HER2 expression the HR was 1.95 (CI 1.04–3.68). In METABRIC the HR was 1.26 (CI 1.00–1.57) and after adjusting for HER2 expression it was HR = 1.06 (CI 0.84–1.34).

To analyze whether VMP1 mRNA status could predict the outcome of BC patients, survival analyses were performed. Breast cancer specific survival (BCSS) was used rather than OS, which may be due to other diseases in addition to BC. Cohort 1 BC patients with tumors expressing high VMP1 mRNA level had shorter BCSS than patients with normal level VMP1 mRNA (log rank p = 0.0045, Fig 3A). The median time of BCSS was 3.75 years for high and 13.22 years for normal VMP1 mRNA, respectively. The HR was 2.31 (CI 1.27–4.18). In the METABRIC cohort high VMP1 mRNA associated with shorter BCSS (log rank p = 0.032) with median survival at 21.7 years with high VMP1 versus 23.5 years for normal VMP1 (Fig 3D). The HR was 1.26 (CI 1.02–1.57). There was not an association between high VMP1 mRNA and BCSS in cohort 2 (log rank p = 0.49 Fig 3B) or in the cohort from TCGA (log rank p = 0.12, Fig 3C). Because VMP1 is necessary for the initial steps of autophagy [45] and autophagy is high in metastatic tumors [46], the effect of high VMP1 levels on distant recurrence free survival (DRFS) was analyzed in the two cohorts for which there were data. High VMP1 was significantly associated with shorter DRFS in cohort 1 (log rank p = 0.0017; HR = 2.54, CI 1.39–4.66) (Fig 4A) as well as METABRIC (log rank p = 0.041; HR = 1.26, CI 1.00–1.57) (Fig 4B). Since HER2 is a potent oncogene and VMP1 was most highly expressed in HER2 positive tumors, the possibility remained that HER2 could be confounding the effect of high VMP1 on survival. Taking into account the effect of HER2 on DRFS revealed that in cohort 1 HR was reduced to 1.95 but it was still significant (CI 1.04–3.68) whereas in METABRIC the HR was no longer significant (HR 1.06, CI 0.84–1.34). This suggests that VMP1 mediates some of the effect on survival in cohort 1 but in METABRIC it was due to HER2. It would be ideal to analyze the association of VMP1 with survival in a large HER2 positive cohort that has not received trastuzumab or another treatment directed against HER2. The patients in cohort 1 and METABRIC did not receive trastuzumab but only the METABRIC dataset had enough tumors to attempt an analysis of BCSS and DRFS in HER2 positive tumors (n = 220). In the METABRIC/HER2 positive cohort high VMP1 was not significantly associated with shorter BCSS (35 vs 185 tumors with high versus low VMP1 mRNA, log rank p = 0.29) but the association was suggestive when analyzed for DRFS (log rank p = 0.085) (S4 Fig). Even though HER2 is a confounder, there appears to be an effect on survival by VMP1 albeit weak (cohort 1). VMP1 can be activated by HER2 through the PI3K/AKT pathway via GLI3-p300 [47] and independent of HER2 e.g. by the hypoxia induced factor HIF1α [48]. Further analyses in cell based systems are necessary to understand how VMP1 contributes to BC progression.

## Discussion

This study describes how fusion genes were used as a tool to identify potential new BC genes with a role in breast tumor development. *VMP1* was the strongest candidate based on our selection criteria. VMP1 mRNA was most highly expressed in HER2 positive tumors, and the results suggest that high VMP1 mRNA may signal worse prognosis for BC patients, most likely in those with HER2 positive tumors.

VMP1 mRNA levels were higher in HER2 positive tumors than HER2 negative tumors in all cohorts analyzed. However, high VMP1 mRNA levels were associated with shorter BCSS in cohort 1 and METABRIC but not in cohort 2 and TCGA. The discrepancy may be due to the extended period of tumor collection and as a result different treatments and the introduction of new drugs during the period. It is tempting to speculate that the differences in the survival analyses between cohorts hinges on trastuzumab because diagnoses of the patients in cohort 1, 1987–2003, preceded the approval of trastuzumab in Iceland, while the diagnoses in cohort 2, 2003–2007, succeeded it, and VMP1 only associated with survival in the former cohort. Furthermore, the patients in the METABRIC cohort, where high VMP1 level associated with shorter BCSS, did not receive trastuzumab [26]. The BC patients in the TCGA cohort were diagnosed over an extended period, 1987–2013, that probably included different types of treatment. However, when HER2 expression was taken into account, the effect on survival was only significant in cohort 1 but not in METABRIC. This was also true when DRFS was analyzed. The discrepancy could be due to different treatments that the patients in these cohorts received, treatments that can have confounding effects on the survival analyses. The four cohorts were diagnosed over extended time periods and as a result they obtained varied drug treatments. Adjusting for all of them in a survival analysis is complex because a subgroup of patients could be selected inadvertently. E.g., in one of the cohorts the patients who were lymph node negative with ER positive tumors received no chemotherapy while the lymph node positive patients with ER negative tumors received chemotherapy. Also, breaking the cohorts down according to drug treatments would reduce the numbers in the cohorts resulting in less power. Therefore, we did not include drug treatments in the analyses. However, they may explain the different results in the cohorts. Also, we performed tumor microarrays to determine whether the VMP1 mRNA levels reflected the protein levels, but correlation of mRNA and protein expression could not be assessed due to background staining with the anti-VMP1 antibody.

VMP1 is a transmembrane protein that is associated with the endoplasmic reticulum, Golgi and intracellular vesicles [49]. VMP1 is important for cellular membrane biology as lack of the protein results in defects in endosome trafficking and Golgi morphology [50]. It also has a role in cell adhesion [51], early autophagosome formation [45] and it controls contact between the endoplasmic reticulum and the isolation membranes that precede the formation of the autophagosome [52]. Domains within the protein appear to be highly conserved between species, even bacteria [53]. VMP1s role in BC is not well known. In ovarian tumors VMP1 has been shown to be highly expressed promoting proliferation and metastasis [49] while in colorectal and hepatocellular cancer cells high levels of VMP1 decrease proliferation, invasion and metastasis [54, 55]. This discordance could be due to tumor type but it may also be due to VMP1s role in autophagy. Autophagy has been suggested to act as a tumor suppressor or tumor promoter depending on context [56], and its activity fluctuates during tumor development [46]. VMP1 interacts with the autophagy regulator BECN1 [57], whose interaction with HER2 inhibits autophagy [58]. In pancreatic cells, VMP1-induces autophagy and the KRAS^G12D^ mutation co-operates to promote the formation of pancreatic ductal adenocarcinoma [59]. Hypoxia inducible factors (HIFs) are activated in regions of rapidly growing tumors that are often poorly oxygenated. HIF1α expression increases VMP1-induced autophagy that results in less cell death in response to photodynamic therapy [48]. HER2 uses the hypoxia system as it regulates HIF2α under normoxic and hypoxic conditions to upregulate hypoxia genes that help the tumor to survive [60]. Thus, VMP1 could support tumor progression at various points, both independent and dependent on HER2.

ERBB2, at 17q12, is amplified in 15% of breast tumors [61]. Genes that are co-amplified with ERBB2 can result in resistance to anti-HER2 therapy. Genes within the 17q12-21 locus are frequently co-amplified with ERBB2, and some of them, like GRB7, have been shown to co-operate with HER2 [62]. Amplification or expression of *TOP2A* is an indicator of worse prognosis in BC patients [63]. It expresses topoisomerase IIα, which is a target of anthracycline. *TOP2A* has been suggested as a biomarker for treatment in HER2 positive BC even though further research is needed [64]. 17q23, where *VMP1* resides, is amplified in 20% of *ERBB2* amplified tumors [23, 40, 44]. A recent study demonstrated that overexpression of only PPMD1 or miR21 from the 17q23 locus co-operated with HER2 to induce growth in soft agar in murine mammary tumor virus cells expressing HER2 (MMTV-ErbB2) [65]. In addition these genes increased resistance to therapy targeting HER2 but targeting HER2 and PPM1D and/or miR21 reduced the tumor burden of the cells. Neither PPM1D nor miR21 abolished the effect of high VMP1 mRNA on survival. The induction of autophagy by VMP1 may be important for the development of drug resistance [66]. Chemotherapy can trigger autophagy [67], which has been shown to contribute to the development of resistance to drugs, including HER2 blockers [68, 69] and tamoxifen [70, 71]. Thus, identifying genes that induce resistance in HER2 positive tumors can benefit patients in the form of additional therapies.

## Conclusions

Taken together, the data presented suggest that high VMP1 expression may be a marker of poor prognosis in BC, particularly in HER2 positive breast tumors. Since VMP1 is important for autophagosome formation, HER2 positive tumors with high VMP1 may more readily initiate autophagy, which provides building blocks for replication and survival, and therefore the patients could be more prone to relapse. Further studies are needed in cell based systems to elucidate the role of VMP1 in breast tumor development.

## Supporting information

S1 Fig Location of the probes within VMP1. (PDF)

S2 Fig The sequenced junction of fusion gene *RPS6KB1-VMP1*. (PDF)

S3 Fig VMP1 mRNA is higher in breast tumors than normal breast tissue in cohorts 1 and 2. (PDF)

S4 Fig A suggestive association was observed between high VMP1 mRNA and DRFS in METABRIC/HER2 positive patients. (PDF)

S1 Table Patient characteristics of cohort 2. (PDF)

S2 Table Amplification and correlation between DNA and mRNA of the gene partners that constitute the fusion genes. (PDF)

S3 Table The effect of VMP1 on overall survival in TCGA was not attenuated by RPS6KB1, PPM1D and miR21. (PDF)

S4 Table Correlation of VMP1 mRNA with clinicopathological characteristics of breast tumors in cohort 2. (PDF)

S5 Table Correlation of VMP1 mRNA with clinicopathological characteristics of breast tumors in TCGA. (PDF)

S6 Table Correlation of VMP1 mRNA with clinicopathological characteristics of breast tumors in METABRIC. (PDF)
