# Supplementary material for: High expression of the vacuole membrane protein 1 (VMP1) is a potential marker of poor prognosis in HER2 positive breast cancer
Source: PLoS One. 2019 Aug 23;14(8):e0221413. doi: 10.1371/journal.pone.0221413 (PMC6707546; doi:10.1371/journal.pone.0221413)
Supplement: S1 Fig — (PDF) [file pone.0221413.s001.pdf]

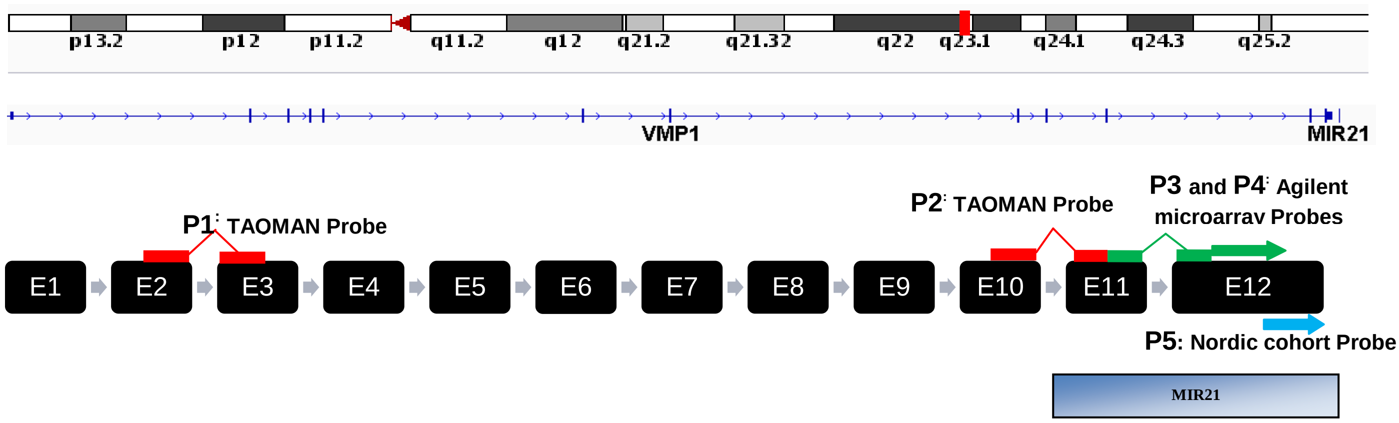

S1 Fig. Location of the probes within VMP1. The schematic at top shows chromosome 17 with a red square that indicates the location of the *VMP1* gene. Below is a stick diagram of the exons within *VMP1* as well as the mature sequence of MIR-21. In the box diagram are shown the exons and the probes used to measure VMP1 mRNA levels. Probe P1 denotes the Taqman probe that spans exons 2 and 3, which was used in cohort 1 to check if MIR21 influenced the measurement of VMP1 levels. P2 denotes the Taqman probe that spans exons 10 and 11, which was used in cohorts 1 and 2. P3 and P4 denote the microarray probes from The Cancer Genome Atlas (TCGA) and the METABRIC cohorts. P5 shows the location of the microarray probe in the Nordic cohort. The box underneath labeled MIR21 shows the position of the MIR21 gene. Note that the mature sequence of MIR21 is telomeric to the VMP1 gene.
