# Supplementary material for: High expression of the vacuole membrane protein 1 (VMP1) is a potential marker of poor prognosis in HER2 positive breast cancer
Source: PLoS One. 2019 Aug 23;14(8):e0221413. doi: 10.1371/journal.pone.0221413 (PMC6707546; doi:10.1371/journal.pone.0221413)
Supplement: S2 Fig — (PDF) [file pone.0221413.s002.pdf]

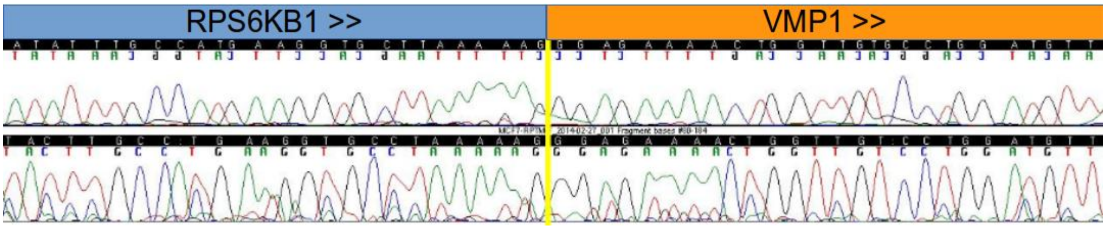

S2 Fig. The sequenced junction of the fusion transcript *RPS6KB1-VMP1*. The sequencing results for *RPS6KB1-VMP1* in the MCF7 breast cancer cell line. The junction site is indicated with a yellow line. The fusion sequence predicted by SOAPfuse is the sequence with white letters on black background (RPS6KB1–CCATGAAGGTGCTTAAAAAG**:** GGAGAAAACTGGTTGTCCTG – VMP1. The chromatogram on top shows the sequence that was obtained using the reverse primer while the lower chromatogram shows the sequence obtained with the forward primer. The junctions of the five fusion genes that passed the criteria were sequenced. The junction of *RPS6KB1-VMP1* is shown here as an example.
