# Supplementary material for: High expression of the vacuole membrane protein 1 (VMP1) is a potential marker of poor prognosis in HER2 positive breast cancer
Source: PLoS One. 2019 Aug 23;14(8):e0221413. doi: 10.1371/journal.pone.0221413 (PMC6707546; doi:10.1371/journal.pone.0221413)
Supplement: S3 Fig — (PDF) [file pone.0221413.s003.pdf]

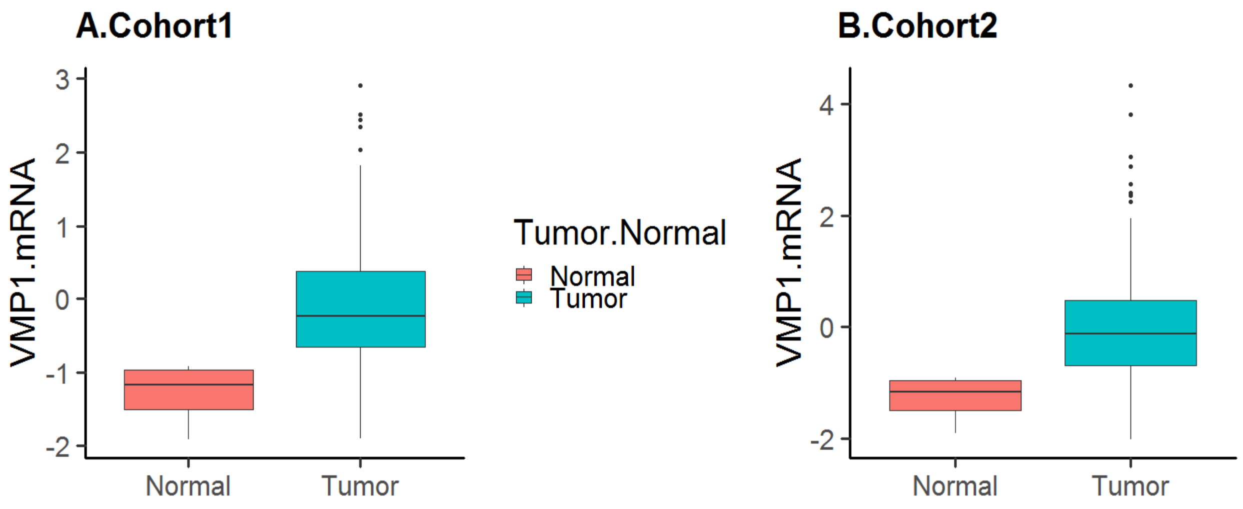

S3 Fig. VMP1 mRNA is higher in breast tumors than normal breast tissue in cohorts 1 and 2. The levels of VMP1 mRNA were compared between 6 normal breast tissue samples and the tumors from A) cohort 1 (n = 141) and B) cohort 2 (n = 277). The mRNA was measured with Taqman Gene Expression Assays spanning exons 10-11 (E10-11). VMP1 expression was calculated relative to the reference gene TBP: $2^{-(\text{mean Ct target} - \text{mean Ct reference})}$. The values were transformed with log2 to normalize the data, which were then used to compare the difference in expression with a Student t-test in R. The p-values were 0.0002 and 0.0003 for cohorts 1 and 2, respectively.
