# Supplementary material for: High expression of the vacuole membrane protein 1 (VMP1) is a potential marker of poor prognosis in HER2 positive breast cancer
Source: PLoS One. 2019 Aug 23;14(8):e0221413. doi: 10.1371/journal.pone.0221413 (PMC6707546; doi:10.1371/journal.pone.0221413)
Supplement: S4 Fig — (PDF) [file pone.0221413.s004.pdf]

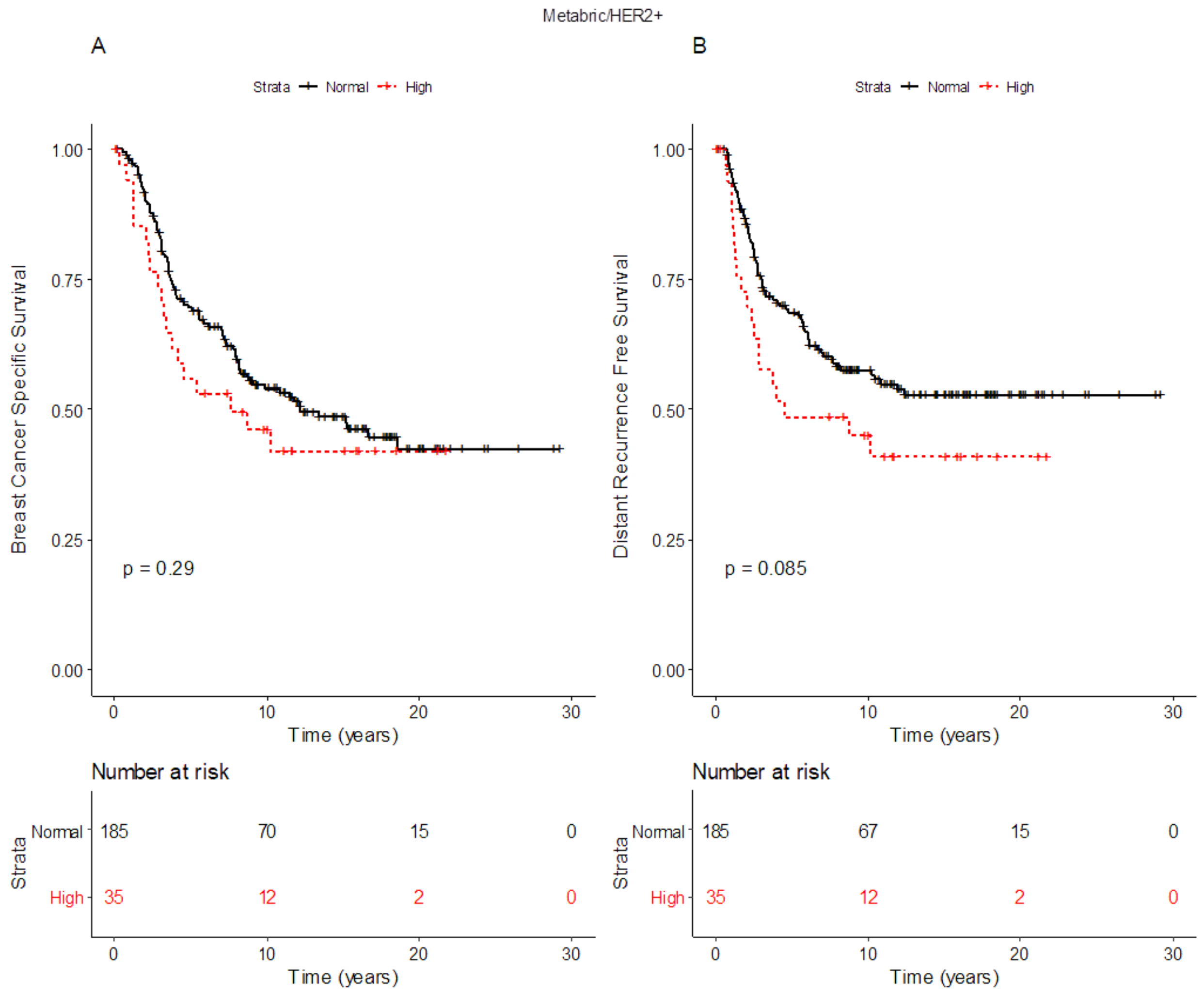

S4 Fig. A suggestive correlation was observed between high VMP1 mRNA and DRFS in METABRIC/HER2 positive patients. A) Breast cancer specific survival (BCSS) and B) distant recurrence free survival (DRFS) was analyzed in HER2 positive breast cancer patients from the METABRIC cohort. The patients were divided into two groups according to VMP1 mRNA levels: tumors expressing high VMP1 mRNA (high ≥ mean + 1 SD) and normal VMP1 (normal < mean + 1 SD). The log rank p-values are indicated in the figures. The number of patients at risk is shown below the graphs at the indicated time points.
