# Supplementary material for: High expression of the vacuole membrane protein 1 (VMP1) is a potential marker of poor prognosis in HER2 positive breast cancer
Source: PLoS One. 2019 Aug 23;14(8):e0221413. doi: 10.1371/journal.pone.0221413 (PMC6707546; doi:10.1371/journal.pone.0221413)
Supplement: S1 Table — (PDF) [file pone.0221413.s005.pdf]

S1 Table: Patient characteristics of cohort 2.

Characteristic	n=277 (%)
Age (year)	
median (range)	60 (27-97)
Estrogen receptor	
positive	194 (70)
negative	77 (28)
unknown	6 (2)
Progesterone receptor	
positive	176 (63.5)
negative	94 (34)
unknown	7 (2.5)
HER2 status	
positive	55 (20)
negative	217 (78)
unknown	5 (2)
Tumor size (mm)	
>20 mm	194 (70)
≤20 mm	82 (29.6)
unknown	1 (0.4)
Histological type	
IDC	231(83)
ILC	30 (11)
other	16 (6)
Nodal status	
positive	146 (53)
negative	101 (36)
unknown	30 (11)
Histological grade	
1	31 (11)
2	124 (45)
3	107 (39)
unknown	15 (5)
Metastasis	
positive	65 (23.5)
negative	210 (75.8)
unknown	2 (0.7)
