# Supplementary material for: High expression of the vacuole membrane protein 1 (VMP1) is a potential marker of poor prognosis in HER2 positive breast cancer
Source: PLoS One. 2019 Aug 23;14(8):e0221413. doi: 10.1371/journal.pone.0221413 (PMC6707546; doi:10.1371/journal.pone.0221413)
Supplement: S2 Table — (PDF) [file pone.0221413.s006.pdf]

S2 Table: Amplification and correlation between DNA and mRNA of the gene partners that constitute the fusion genes.

Gene	Amplification (%)		Correlation (r)	
	Nordic	TCGA	Nordic	TCGA
ANK3	0.29	2.8	0.20*	0.39*
CCDC170	0.6	1.9	0.07	0.03
CCDC6	0.9	1.5	0.52*	0.66*
ESR1	0.29	2.5	0.04	-0.05
GATAD2B	3.07	12.7	0.55*	0.54*
ITGB6	0	0.9	-0.01	0.15*
NUP210L	0.92	12.9	0.01	0.16*
RBMS1	0.93	0.6	NA	0.31*
RPS6KB1	6.97	10.9	NA	0.83*
VMP1	7.4	10.7	0.45*	0.70*

Listed above are the ten genes that constitute the five fusion genes that were found in common between breast tumors and breast cancer cell lines. The number of tumors in each cohort that had both DNA and mRNA as measured by comparative genomic hybridization (CGH) and microarrays, respectively, are: Nordic tumors, n = 337 and TCGA = 421. Amplification denotes the number of tumors that carried amplification of the gene in these cohorts. The Pearson correlation (r) was calculated between DNA and mRNA. *Denotes a significant result, $p < 0.05$.
