# Supplementary material for: High expression of the vacuole membrane protein 1 (VMP1) is a potential marker of poor prognosis in HER2 positive breast cancer
Source: PLoS One. 2019 Aug 23;14(8):e0221413. doi: 10.1371/journal.pone.0221413 (PMC6707546; doi:10.1371/journal.pone.0221413)
Supplement: S3 Table — (PDF) [file pone.0221413.s007.pdf]

S3 Table. The effect of VMP1 on overall survival in TCGA was not attenuated by RPS6KB1, PPM1D and miR21.

17q23 amplicon genes	HR	95% CI	p-value
$VMP1^{high}$	2.10	1.09 – 4.04	0.02
$VMP1^{high}$ + $RPS6KB1^{high}$	3.24	1.54 – 6.82	0.001
$VMP1^{high}$ + $PPMD1^{high}$	2.21	1.02 – 4.82	0.04
$VMP1^{high}$ + $miR21^{high}$	2.97	1.15 – 7.72	0.02

The table shows the median and the 25th and 75th percentiles. The p-value was calculated with normalized Z-scores (A_23_P129935) using a t-test or ANOVA. *Significant difference $p < 0.05$.

The table shows the influence of RPS6KB1, PPM1D and miR21 on the effect of VMP1 on overall survival in the TCGA cohort. The mRNA and microRNA levels were divided into high (mean + 1 SD) and normal (mean – 1 SD). The hazard ratio (HR) and 95% confidence interval (CI) from a multivariate Cox regression analyses are shown.
