# Supplementary material for: High expression of the vacuole membrane protein 1 (VMP1) is a potential marker of poor prognosis in HER2 positive breast cancer
Source: PLoS One. 2019 Aug 23;14(8):e0221413. doi: 10.1371/journal.pone.0221413 (PMC6707546; doi:10.1371/journal.pone.0221413)
Supplement: S4 Table — (PDF) [file pone.0221413.s008.pdf]

S4 Table. Correlation of VMP1 mRNA with clinicopathological characteristics of breast tumors in cohort 2

Characteristic	n=277	VMP1 mRNA level, median (25th , 75th)	p-value
Age			
≥ 50	213	-0.10 (-0.66, 0.42)	0.7
< 50	64	-0.32 (-0.92, 0.68)	
Estrogen receptor			
positive	194	-0.15 (-0.67, 0.45)	0.9
negative	77	-0.08 (-0.92, 0.42)	
unknown	6		
Progesterone receptor			
positive	176	-0.21 (-0.70, 0.41)	0.4
negative	94	-0.08 (-0.80, 0.56)	
unknown	7		
HER2 status			
positive	55	0.27 (-0.39, 0.98)	0.004*
negative	217	-0.17 (-0.80, 0.39)	
unknown	5		
Receptors ER and ERBB2			
ER neg and HER2 neg	38	-0.46 (-1.19, 0.10)	0.001*
ER neg and HER2 pos	23	0.27 (0.39, 1.02)	
ER pos and HER2 neg	133	-0.36 (-0.84, 0.30)	
ER pos and HER2 pos	18	0.08 (-0.51, 0.93)	
Tumor size (mm)			
> 20	194	-0.08 (-0.64, 0.50)	0.05
≤ 20	82	-0.26 (-0.81, 0.32)	
unknown	1		
Histological type			
IDC	231	-0.09 (-0.76, 0.51)	0.3
ILC	30	-0.41 (-0.72, 0.06)	
other	16	-0.11 (-0.64, 0.33)	
Nodal status			
positive	146	-0.22 (-0.74, 0.47)	0.6
negative	101	-0.07 (-0.77, 0.50)	
unknown	30		
Histological grade			
1	31	-0.35 (-0.82, 0.08)	0.04*
2	124	-0.11 (-0.64, 0.37)	
3	107	-0.07 (-0.79, 0.83)	
unknown	15		
Metastasis			
positive	65	0.00 (-0.76, 0.5)	0.7
negative	210	-0.12 (- 0.65, 0.45)	
unknown	2		

The table shows the median and the 25th and 75th percentiles. The p-value was calculated with $\log_2$ normalized data using a t-test or ANOVA. *Significant difference $p < 0.05$.
