# Supplementary material for: High expression of the vacuole membrane protein 1 (VMP1) is a potential marker of poor prognosis in HER2 positive breast cancer
Source: PLoS One. 2019 Aug 23;14(8):e0221413. doi: 10.1371/journal.pone.0221413 (PMC6707546; doi:10.1371/journal.pone.0221413)
Supplement: S5 Table — (PDF) [file pone.0221413.s009.pdf]

S5 Table. Correlation of VMP1 mRNA with clinicopathological characteristics of breast tumors in TCGA.

Characteristic	n= 421	VMP1 mRNA level, median (25th , 75th)	p-value
Age			
≥ 50	125	0.16(-0.49, 1.18)	0.6
< 50	296	0.23 (-0.86, 1.29)	
Estrogen receptor			
positive	323	0.39 (-0.41, 1.34)	$7x10^{-6*}$
negative	92	-0.43 (-0.99, 0.28)	
unknown	6		
Progesterone receptor			0.008*
positive	273	0.42 (-0.33, 1.33)	
negative	141	-0.31 (-0.87, 0.55)	
unknown	7		
HER2 status			0.003^{*}
positive	73	0.61 (-0.30, 1.85)	
negative	215	0.14 (-0.57, 0.96)	
unknown	133		
Histological type			0.8
IDC	360	0.22 (-0.50, 1.26)	
ILC	34	0.45 (-0.51, 1.22)	
unknown	27		
Nodal status			0.01*
positive	206	0.07 (-0.69, 1.08)	
negative	206	0.35 (-0.49, 1.32)	
unknown	9		
Subtype			$2x10^{-12*}$
Basal	77	-0.58 (-1.17, -0.22)	
ERBB2	40	0.58 (-0.30, 2.02)	
Luminal A	210	0.38 (-0.47, 1.23)	
Luminal B	89	0.68 (-0.30, 1.92)	
Normal-like	4	0.36 (-0.29, 0.68)	
unknown	1		

The table shows the median and the 25th and 75th percentiles. The p-value was calculated with normalized Z-scores (A_23_P129935) using a t-test or ANOVA. *Significant difference $p < 0.05$.
