# Supplementary material for: High expression of the vacuole membrane protein 1 (VMP1) is a potential marker of poor prognosis in HER2 positive breast cancer
Source: PLoS One. 2019 Aug 23;14(8):e0221413. doi: 10.1371/journal.pone.0221413 (PMC6707546; doi:10.1371/journal.pone.0221413)
Supplement: S6 Table — (PDF) [file pone.0221413.s010.pdf]

S6 Table. Correlation of VMP1 mRNA with clinicopathological characteristics of breast tumors in METABRIC.

Characteristic	n=1904	VMP1 mRNA level, median (25th, 75th)	p-value
Age			
≥ 50	1493	0.18 (-0.52, 1.00)	0.7
< 50	411	0.07 (-0.51, 0.97)	
Estrogen receptor			
positive	1459	0.24 (-0.48, 1.04)	0.01*
negative	445	-0.04 (-0.69, 0.87)	
Progesterone receptor			
positive	1009	0.16 (-0.51, 0.97)	0.5
negative	895	0.15 (-0.54, 1.05)	
HER2 status			
positive	236	0.97 (0.14, 2.20)	$<2x10^{-16}$*
negative	1668	0.07 (-0.61, 0.85)	
Histological type			
IDC	1502	0.18 (-0.51, 1.05)	0.02*
ILC	141	-0.04 (-0.76, 0.73)	
Other	261	0.15 (-0.5, 0.99)	
Subtype			
Basal	199	-0.38 (-1.02, 0.37)	$<2x10^{-16}$*
ERBB2	220	0.66 (-0.09, 1.65)	
Luminal A	679	0.09 (-0.53, 0.78)	
Luminal B	461	0.47 (-0.43, 1.26)	
Normal-like	140	0.22 (-0.62, 1.02)	
claudin-low	199	0.09 (-0.52, 0.91)	
unknown	6		

The table shows the median and the 25th and 75th percentiles. The p-value was calculated with normalized Z-scores (Illumina Human v3 microarray) using a t-test or ANOVA. *Significant difference $p < 0.05$.
